# Design and Rationale for the Deep South Interactive Voice Response System–Supported Active Lifestyle Study: Protocol for a Randomized Controlled Trial

**DOI:** 10.2196/29245

**Published:** 2021-05-25

**Authors:** Nashira I Brown, Mary Anne Powell, Monica Baskin, Robert Oster, Wendy Demark-Wahnefried, Claudia Hardy, Maria Pisu, Mohanraj Thirumalai, Sh'Nese Townsend, Whitney N Neal, Laura Q Rogers, Dori Pekmezi

**Affiliations:** 1 Department of Health Behavior University of Alabama at Birmingham Birmingham, AL United States; 2 Department of Medicine Division of Preventive Medicine University of Alabama at Birmingham Birmingham, AL United States; 3 O’Neal Comprehensive Cancer Center at UAB Birmingham, AL United States; 4 Department of Nutrition Sciences University of Alabama at Birmingham Birmingham, AL United States; 5 Health Services Administration University of Alabama at Birmingham Birmingham, AL United States

**Keywords:** exercise, health disparities, interactive voice response system, physical activity, rural health, telehealth

## Abstract

**Background:**

The rates of physical inactivity and related cancer incidence and mortality are disproportionately high in the Deep South region in the United States, a rural, medically underserved region with a large African American population compared with the rest of the nation. Given this region’s lower rates of literacy and internet access, interactive voice response (IVR) system–automated telephone-based interventions have the potential to help overcome physical activity intervention barriers (literacy, internet access, costs, and transportation) but have yet to be extended to rural, underserved populations, such as in the Deep South. Thus, extensive formative research is being conducted to develop and beta test the Deep South IVR System–Supported Active Lifestyle intervention in preparation for dissemination in rural Alabama counties.

**Objective:**

This paper aims to describe the design and rationale of an ongoing efficacy trial of the Deep South IVR System–Supported Active Lifestyle intervention.

**Methods:**

A two-arm randomized controlled trial will be conducted to compare a 12-month physical activity intervention versus a wait-list control condition in 240 underactive adults from 6 rural Alabama counties. The Deep South IVR System–Supported Active Lifestyle intervention is based on the Social Cognitive Theory and includes IVR-automated physical activity–related phone counseling (daily in months 0-3, twice weekly in months 4-6, and weekly in months 7-12) and support from local rural county coordinators with the University of Alabama O’Neal Comprehensive Cancer Center Community Outreach and Engagement Office. The primary outcome is weekly minutes of moderate- to vigorous-intensity physical activity (7-day physical activity recall; accelerometry) at baseline, 6 months, 12 months, and 18 months. Rural Active Living Assessments will be conducted in each rural county to assess walkability, assess recreational amenities, and inform future environment and policy efforts.

**Results:**

This study was funded in March 2019 and approved by the institutional review board of the University of Alabama at Birmingham in April 2019. As of February 2020, start-up activities (hiring and training staff and purchasing supplies) were completed. Study recruitment and assessments began in September 2020 and are ongoing. As of February 2021, a total of 43 participants have been enrolled in Dallas County, 42 in Sumter County, and 51 in Greene County.

**Conclusions:**

IVR-supported phone counseling has great potential for addressing physical activity barriers (eg, culture, literacy, cost, or transportation) and reducing related rural health disparities in this region.

**Trial Registration:**

ClinicalTrials.gov NCT03903874; https://clinicaltrials.gov/ct2/show/NCT03903874.

**International Registered Report Identifier (IRRID):**

DERR1-10.2196/29245

## Introduction

### Background

Despite the health benefits associated with physical activity, the levels of engagement in regular physical activity remain low in the United States, especially in the Deep South (ie, Alabama, Georgia, Louisiana, Mississippi, and South Carolina) [1]. Furthermore, the rates of related cancer incidence and mortality are generally higher in this underserved (rural, mostly minority) region [2] compared with the national average. Physical activity interventions are needed to address barriers related to transportation, finances, culture, and low literacy and education in the Deep South [3].

Telephone-based strategies do not require frequent clinic visits [4], literacy, or expensive technology and have led to substantial increases in physical activity in past studies [5]. However, there has been a paucity of research in this area among underserved (rural, minority) populations [6-10]. Moreover, most telephone-based interventions to date have involved counseling from health care providers or research staff [4] but can be automated with interactive voice response (IVR) systems for improved reach and reduced cost in resource-strapped rural counties.

In response, our research team developed a tailored, IVR-supported physical activity intervention for cancer risk reduction in the Deep South. The development of the Deep South Interactive Voice Response System–Supported Active Lifestyle (DIAL) intervention was guided by extensive formative research (11 focus groups with African American community health advisors and community members) [11] on physical activity intervention preferences and barriers in our target population. Results from the subsequent pilot randomized controlled trial with 63 participants supported the feasibility and acceptability of the DIAL intervention. At 12 weeks, retention (88.9%) and participant satisfaction (71.4%) were high. Furthermore, intervention participants reported greater increases in moderate-to-vigorous physical activity than control participants from baseline to 12 weeks (median change of 47.5 vs 5 min per week, respectively) and statistically significant improvements in physical activity self-regulation and social support [12]. Pilot trial findings and participant feedback guided intervention refinement (providing more accountability and encouragement) in preparation for scale-up.

### Objectives

Given the promise shown in the pilot study, this study involves an amply powered randomized controlled trial (N=240) of the refined DIAL intervention in 6 rural counties in Alabama. To our knowledge, this study is the first to implement an IVR system–supported physical activity intervention in rural, mostly minority populations. The primary aim of this study is to test the efficacy of the DIAL intervention versus a wait-list control. We hypothesize that the participants receiving the DIAL intervention will report significantly greater increases in moderate-to-vigorous physical activity based on the 7-day physical activity recall (PAR) interviews and accelerometers at 6 months compared with participants randomized to a 6-month wait-list control arm.

The exploratory aims are to examine (1) changes in moderate-to-vigorous physical activity at 12 months and 18 months to assess long-term maintenance in the intervention arm and ascertain replicability of intervention effects in the wait-list control arm; (2) intervention effects on physical performance, anthropometrics, and psychosocial variables; (3) cost-effectiveness; (4) potential mediators (Social Cognitive Theory [SCT] constructs directly targeted by the intervention) and moderators (education and neighborhood and environmental features) of treatment efficacy; and (5) potential barriers to or facilitators of widespread implementation of the DIAL intervention in the rural Deep South, a region spreading across central Alabama and Mississippi that is known for both its rich black soil and high population of non-Hispanic Black individuals [13].

## Methods

### Recruitment and Eligibility Criteria

A total of 240 participants will be recruited from 6 rural Alabama counties (Hale, Choctaw, Greene, Marengo, Dallas, and Sumter) by local rural county coordinators from the University of Alabama (UAB) O’Neal Comprehensive Cancer Center Office of Community Outreach and Engagement (O’Neal CCC COE). The local rural county coordinators are well-respected and trusted individuals who reside in the targeted rural communities and collectively have over 50 years of experience working in their communities, implementing various community outreach and research programs.

The local rural county coordinators received training on study protocols from the DIAL program manager and the O’Neal CCC COE program director and program manager. The training included a comprehensive project overview that covered project goals with an emphasis on the county coordinators’ role, participant recruitment, and data collection during assessments. Coordinators were provided with training manuals that included project protocols, assessment tools, and other forms.

Primary forms of recruitment include local newspapers and radio advertisements, study flyers, and word-of-mouth. The trial was approved by the UAB Institutional Review Board (IRB) and registered with ClinicalTrials.gov (NCT03903874). Although IRB approval was obtained for recruitment via social media, this option is yet to be used in this study. Recruitment occurs on a rolling basis, staggered by county. Approximately 40 participants will be recruited from each of the 6 counties over a 30-month period.

Potential participants are screened for eligibility via telephone by the research staff. The inclusion criteria are as follows: (1) ≥18 years of age; (2) inactive (ie, reporting less than 60 min of moderate-to-vigorous physical activity per week); and (3) residing or working in one of the participating counties. Individuals will be excluded on the basis of the following criteria: (1) presence of a medical condition that could make unsupervised physical activity unsafe (ie, history of heart disease, stroke, or orthopedic conditions that limit mobility based on the Physical Activity Readiness Questionnaire [14]); (2) plans to move from the area in the next 18 months; (3) unable to speak or read English; (4) unwilling to be randomized to either the DIAL intervention or wait-list control arm and adhere to the respective protocols; and/or (5) lack of access to a telephone.

### Informational Session

Once screened for eligibility, the participants will be scheduled for an information session via Zoom (Zoom Video Communications, Inc). The program manager will provide them with an overview of study protocols, participant expectations, research staff expectations, and informed consent and then schedule baseline assessments.

### Baseline Assessments

**Figure 1:**
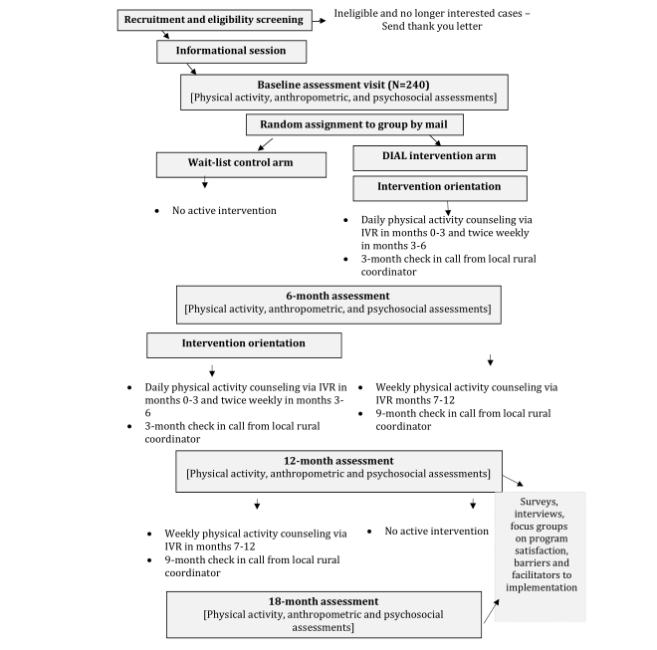
Study flow. DIAL: Deep South Interactive Voice Response System–Supported Active Lifestyle. IVR: interactive voice response.

Baseline assessments occur at convenient local community locations (eg, church halls, local high schools, or county health departments). At this visit, participants will complete anthropometric measurements (ie, height, weight, and waist circumference), surveys, and the Two Minute Step Test (see the *Outcomes* section for further details). Participants will also receive an accelerometer with instructions to wear the device for 7 days and scheduled a 7-day PAR telephone interview. Figure 1 shows the study flow.

### Randomization

Participants will be randomly assigned to the DIAL intervention condition after completing the baseline assessment using a stratified block randomization scheme, stratifying by county, and using a block size of 4. The randomization lists are computer generated using SAS (version 9.4). Participants will receive group assignment information via mail along with a pedometer and/or FitBit Inspire, if assigned to the DIAL intervention arm.

#### DIAL Orientation

During the intervention orientation, participants will be instructed to wear their physical activity tracker daily during waking hours for the next 12 months and learn how to complete the IVR system calls. Personal identification numbers are provided to confirm the identity and preserve confidentiality. IVR system calls will be scheduled per the participants’ preferred time.

The staff encourage participants to gradually increase their physical activity from week to week until reaching the national guidelines of 150 minutes per week [15] of moderate-to-vigorous physical activity with an emphasis on safety and injury prevention (moderate-intensity physical activity, stretching, warming up, and cooling down).

#### DIAL Intervention

The 12-month DIAL intervention is based on the SCT, which posits that attitudes and beliefs, physical and social environment, and behaviors mutually influence each other [16]. Key SCT constructs (social support, self-efficacy, self-regulation, outcome expectations, and enjoyment) are targeted through the use of study-provided pedometers (Accusplit, AX2790MV), a FitBit Inspire, and individually tailored physical activity counseling calls [12].

The physical activity counseling calls are automated using the Twilio cloud communications platform and Amazon Polly for narration. Participants will complete calls daily in months 0-3, twice weekly in months 4-6, and weekly in months 7-12. There are three types of calls: physical activity tracking, goal setting, and counseling. The tracking calls allow the participant to report the number of steps and minutes of moderate-to-vigorous physical activity for the previous day. Participants set new step goals in weekly goal-setting calls and reflect on their progress. To encourage incremental increases toward 10,000 steps per day, participants will be asked if they are ready for a challenge, and if so, they will be encouraged to increase their step goal by 250 steps per day for that week. The intentionally modest number of steps was selected as it represents approximately an eighth of a mile and can be easily achieved while walking in place for the duration of the 2- to 3-minute IVR system call.

During the monthly IVR system counseling calls, participants will complete 4 brief SCT surveys (physical activity self-efficacy, enjoyment, outcome expectations, and social support) and receive individualized IVR system feedback based on their responses. Tailored algorithms focus on whether generally high or low levels of each SCT construct were described and whether these values represent improvements from the previous report. For example, for lower self-efficacy scores, participants may receive the message, “You do not sound very sure about your ability to exercise, but it’s better than last time we spoke. Try squeezing in a 10 minute walk this week. Meeting this small goal will help you feel more sure that you can fit physical activity into your life.”

As fresh, evolving IVR system call content will be critical for maintaining participant engagement over 12 months, libraries of rotating message options for greetings, step and moderate-to-vigorous physical activity feedback, goal setting, and SCT counseling were developed. Calls also include new physical activity tips each week on how to get more steps and physical activity benefits (eg, stronger bones and joints, toned muscles, and improved function of the heart, lungs, and other body systems) and addresses barriers (eg, lack of time, lack of support, or negative expectations). Participants will be alerted during the call when new tips are available, for example, “We have a new tip this week to encourage you as you become more active. Check it out at the end of the call!”

Participants will also receive support from their local rural county coordinator, who will call to check in on physical activity motivation and goals at months 3 and 9. Moreover, participants will receive the monthly newsletter, *The Deep South Interactive Voice Response System–Supported Active Lifestyle Intervention Dispatch*, highlighting local physical activity resources (ie, recreational facilities, walking and hiking and biking trails, and playing fields and courts) and opportunities. Also featured in the newsletter are interviews with local rural county coordinators and *selfies* of participants engaging in physical activity in their community.

### Treatment Fidelity

The treatment fidelity plan is based on the National Institutes of Health Behavioral Change Consortium framework [17], which will be implemented with checklists, scripted treatment manuals, audio-recordings of participant encounters, and transcripts of sessions (eg, for intervention orientations and 3- and 9-month local rural county coordinator support calls). The staff will carefully monitor IVR system call completion via weekly generated reports and contact and/or re-engage participants who have reported medical events, nonadherence to pedometer use, and/or moderate-to-vigorous physical activity or who missed two or more scheduled calls.

To identify any *glitches* with automated physical activity–related telephone counseling, communicate these issues to programmers, and ensure appropriate feedback is being provided to the participants, 5 investigators and 6 research staff members began quality control for the IVR system regularly (daily in the first 3 months, biweekly in months 4-6, and weekly in months 7-12, similar to the intervention call schedule) in June 2020.

### Wait-List Arm (Control Arm)

Participants in the wait-list control arm are encouraged to maintain their usual activity levels until completion of the 6-month assessments and then receive the same 12-month DIAL intervention. During the wait-list period, these participants will receive monthly University of Alabama at Birmingham O’Neal Comprehensive Cancer Center Office of Community Outreach and Engagement (UAB O’Neal CCC COE) newsletters and webinar invitations on cancer-related topics other than physical activity (ie, plant-based diet, healthy grocery list, mental health, developing a self-care routine, and cancer prevention).

### Assessments at 6, 12, and 18 Months

At 6, 12, and 18 months, participants will repeat accelerometer protocols, 7-day PAR interviews, anthropometric assessments, Two Minute Step Tests, and psychosocial surveys. Participants will complete additional surveys and exit interviews on program satisfaction at 12 months (for the intervention arm) and 18 months (for the wait-list arm).

### Outcomes

The study measures are listed in Table 1. Outcome measures will be conducted at baseline and at the 3-, 6-, 12-, and 18-month assessments.

**Table 1:** Deep South Interactive Voice Response System–Supported Active Lifestyle study measures.

Outcomes			Baseline		Month 3		Month 6		Month 12		Month 18
**Physical activity**											
	7-day physical activity recall	✓^a^				✓		✓		✓	
	ActiGraph GT3X accelerometers	✓				✓		✓		✓	
	Two Minute Step Test	✓				✓		✓		✓	
**Potential moderators**											
	Anthropometrics (height, weight, or waist circumference)	✓				✓		✓		✓	
	Demographics	✓									
	Rural Active Living Assessment (environment, walkability, or community programs)	✓									
**Potential mediators**											
	Self-regulation scale	✓		✓		✓		✓		✓	
	Social support for exercise scale	✓		✓		✓		✓		✓	
	Outcome expectations scale	✓		✓		✓		✓		✓	
	Physical activity enjoyment scale	✓		✓		✓		✓		✓	
	Walking self-efficacy	✓		✓		✓		✓		✓	
**Cost-effectiveness analysis measures**											
	Health care use	✓				✓		✓			
	EuroQol-5D^b^	✓				✓		✓			
	Intervention participation questions	✓				✓		✓			
**Other**											
	Psychosocial measures	✓				✓		✓		✓	
	Participant satisfaction measure							✓		✓	
	Stakeholder acceptability survey									✓	

^a^Time point of assessment.

^b^EuroQol-5D: EuroQol- 5 Dimension.

#### Primary Outcomes

The main outcome is minutes per week of moderate- to vigorous-intensity physical activity. The 7-day PAR interview is administered by trained research staff, who will contact participants by telephone and ask about the amount, intensity, and types of physical activities over the past 7 days [18,19]. The 7-day PAR has demonstrated reliability, congruent validity, and internal consistency [20-28]. The instrument is sensitive to moderate changes in physical activity over time [29,30] and has been validated by telephone [22]. Moreover, these self-reported data will provide useful insights regarding the specific types of physical activities occurring in rural counties.

ActiGraph GT3X accelerometers are worn continuously for 7 days during waking (on the nondominant hip) and sleeping (on the nondominant wrist) hours at baseline and at 6, 12, and 18 months. This device measures movement, intensity of physical activity, and sleep efficiency, latency, and number of awakenings. The devices have been validated using heart rate telemetry [31] and total energy expenditure [32] and have been shown to provide valid estimates of sleep [33]. A minimum threshold of 1952 counts per minute [32,34] will be used for moderate- to vigorous-intensity physical activity with an epoch of 30 seconds. The minimum valid wear time has been set at 4 days of at least 600 minutes of wear. This objectively measured data will be used to corroborate self-reported PAR data.

#### Secondary Outcomes

The secondary outcomes include physical performance and anthropometrics. Physical performance is assessed with the Two Minute Step Test, in which participants step in place as fast as possible for 2 minutes while lifting the knees to a premeasured height midway between the upper tips of their patella and iliac crest when standing [35]. A score is calculated based on the number of times the right knee meets the marked height, which can be used to estimate the current level of physical function and predict future physical independence [36,37]. Anthropometric measurements include height, weight, and waist circumference. Height will be measured without shoes and with a portable stadiometer (Seca 213). Weight will be measured without shoes and in light clothing with a digital scale (Healthometer, model no: 349KLX) that is zeroed before each measurement. Waist circumference will be measured with a Gulick II tension-controlled tape measure (County Technology, Gary Mills). The tape is positioned around the natural waist, just above the iliac crest. The measurement is recorded to the nearest 0.1 cm upon exhalation.

Psychosocial factors will also be assessed at baseline and at 6, 12, and 18 months using Patient-Reported Outcome Measurement Information System scales for anxiety, depression, fatigue, and sleep disturbance with previously demonstrated validity and reliability (α=.95, .98, .84, and .83, respectively) [38].

SCT measures will be assessed in person at the 4 assessment visits and by mail at 3 months (for mediation analyses). The measures include the 10-item self-regulation scale (α=.78) [39], 13-item social support for exercise scale (α=.61-.91) [40], 9-item outcome expectations scale (α=.89) [41], 18-item physical activity enjoyment scale with high internal consistency and test-retest reliability [42], 10-item walking self-efficacy scale (α=.82) [43], and the 12-item exercise confidence scale (α=.92) [44].

Secondary measures at baseline, 6 months, and 12 months were included in the cost-effectiveness analyses. We will use a health care utilization survey that captures information on physician and emergency room visits and hospitalizations [45]; the EuroQol-5 Dimension, which estimates utility weights to estimate quality-adjusted life years (QALYs) [46]; and a set of questions to measure time spent and expenses related to participation and time devoted to physical activity [46].

An 18-item measure adapted from similar past studies [47,48] will assess participant satisfaction with the DIAL intervention and request suggestions for program improvement at 12 and 18 months. Finally, a similar survey will be administered at 18 months to the rural county coordinators to examine stakeholders’ perspectives on acceptability, barriers to and facilitators of implementation, and sustainability of the DIAL intervention in the Deep South.

The research staff will complete the three components of the Rural Active Living Assessment (RALA) for each county at baseline. This assessment consists of the street segment assessment to evaluate factors such as walkability, safety features, and terrain of individual, specific street segments; the town-wide assessment that examines community characteristics such as population, total area, and the presence of recreation activities; and finally, the program and policy assessment, which identifies community programs and policies that support physical activity [49]. These tools have been successfully used in similar past studies conducted by our research team in the Deep South [11].

### Protocol Changes in Response to Feedback From Community Partners

Modifications to assessment and intervention protocols are often necessary to meet the needs of study populations and occur frequently in response to concerns and guidance from community stakeholders in this study. For example, the originally proposed 12-month wait-list control phase was reduced to 6 months due to stakeholder concerns (UAB O’Neal CCC COE, local rural county coordinators, etc) regarding withholding active intervention for so long and how this might affect participant and community engagement. In addition, we added 3-month SCT surveys to address stakeholders’ concerns and still be able to comment on potential mediators. Participant frustrations with malfunctioning pedometers and enthusiasm and desire for wristbands resulted in offering FitBit options for tracking steps.

As for assessment protocols, several instruments were cut to reduce participant burden. More specifically, short-form versions of social support and exercise and walking self-efficacy were adopted [50,51]. The Patient-Reported Outcome Measurement Information System subscales on anxiety, depression, and fatigue and exercise confidence scales were eliminated. Moreover, incentives for completing assessments were increased from the proposed amount of US $25-US $50 based on UAB O’Neal CCC COE feedback on the time spent completing surveys and incentive amounts from past studies.

### Safety Precautions Related to COVID-19

In response to COVID-19, intervention and assessment protocols were substantially modified for participant and research team safety (and adherence to UAB IRB requirements). The staff are now relying solely on socially distanced recruitment strategies such as posting flyers, newspaper advertisements, emails, text messages, and telephone calls. Rather than attending in-person group information sessions to learn more about the study and complete informed consent, participants initially received a protocol overview by telephone with UAB O’Neal CCC COE county coordinators. However, these brief study descriptions were perhaps less detailed than the longer in-person information sessions and resulted in some participant confusion regarding study protocols and expectations. Thus, a compromise was reached by holding full study information sessions via Zoom (Zoom Video Communications, Inc). To further reduce face-to-face time, we will conduct the informed consent process on the web before the baseline assessments.

During the appointment reminder call and upon arriving for the baseline assessments, participants will complete COVID-19 symptoms and exposure screenings, including two survey items (ie, “Have you had any of the following symptoms in the last 14 days?” “To your knowledge, have you been in close contact with anyone diagnosed with COVID-19 in the last 14 days?”). The temperature of the participants is measured using a contact-free thermometer. If a participant has a temperature of 100.4°F or higher and/or answers *yes* to screening items, their assessment appointment will be postponed, and the participant will be asked to contact their primary care physician.

Baseline assessments still occur in convenient community locations but with social distancing and appropriate personal protective equipment. Participants will be offered masks (if needed) and hand sanitizers. Research staff will wear personal protective equipment and wipe down all surfaces and equipment with antiseptic wipes between each participant contact. Assessment stations (for surveys, anthropometric measurements, accelerometers, and scheduling) will be spaced at least 6 feet apart. Participant appointments will be carefully scheduled throughout the day to avoid crowding. Clear plastic dividers will be set up as needed at assessment stations. To reduce face-to-face assessment time, the 7-day PAR interviews will be conducted by telephone using previously validated protocols [21].

To further reduce exposure, randomization to the DIAL intervention arm and IVR call orientation are no longer provided at local community locations but only by mail and videoconferencing. Participants will receive COVID-19 physical activity safety precaution handouts in their intervention orientation packets, which will encourage them to wear a face mask and distance themselves from others while being physically active. Moreover, we have edited any IVR system counseling messages that might be unsafe or unwise during a pandemic.

County coordinators initially planned to organize community walking groups for interested participants but are now pursuing distance-based approaches to build community support for physical activity. For example, participants are encouraged to text *selfies* of themselves being active in their community to their local rural county coordinators for a prize (ie, resistance bands, water bottles, or sweatbands) and the chance to have their selfie featured in the monthly newsletter. The wait-list control group was originally scheduled to attend in-person monthly *lunch-and-learns* with the county coordinators, but this cancer control education is now provided virtually through webinars.

RALA protocols have also been modified in response to the pandemic. The driver and observer no longer sit adjacent to another in the vehicle. These assessments are now completed in a three-row van or sports utility vehicle to allow both individuals to be positioned at least 6 feet apart for social distancing. We have also reduced the number of designated street segments to decrease the amount of time spent in the vehicle. Moreover, research team members and local rural county coordinators will carefully track the COVID-19–related changes in the available physical activity resources and programming (ie, facility hours and physical activity classes) in these rural counties while conducting RALAs and add two items on COVID-19–related physical activity changes and barriers to the 3-month surveys.

### Data Management

Data were entered into databases created using the Research Electronic Data Capture System. Relational logic checks, such as out-of-range values and internal inconsistencies, will be implemented at the time of initial data entry and will then be assessed periodically to minimize and detect data entry errors. Statistical progress reports will be generated to include the following: (1) the total number of participants screened, consented, and randomized on study entry and completing each follow-up assessment and (2) a summary of demographic and baseline characteristics for comparability between randomization arms. 

### Sample Size Justification

Results from our previous UAB pilot study indicated an increase in minutes of moderate-to-vigorous physical activity among the DIAL intervention group compared with the control group. The SD of this measure was 90 minutes. Assuming a mean difference of 35 (SD 90) minutes in moderate-to-vigorous physical activity between the two groups from baseline to 6 months, a two-tailed two-group *t* test, and a significance level of 5%, we will have 80% power to detect this difference (with an effect size of 0.388) with 105 participants per arm (210 for the study). Assuming a mean change of 35 (SD 90) minutes in moderate-to-vigorous physical activity from baseline to 6 months for the intervention group, a two-tailed paired *t* test, and a significance level of 5%, we will have 80% power to detect this within-group change (with an effect size of 0.276) with 105 participants. Allowing for 15% attrition, we will recruit 240 participants (120 per arm).

### Statistical Analyses

Analyses will be performed on an intent-to-treat basis (ie, participants will be analyzed by the arms to which they were randomized). The characteristics of the study populations will be summarized for each study arm using descriptive statistics, such as means and SD for continuous variables and frequencies and proportions for categorical variables. Unadjusted comparisons of baseline characteristics between study arms and those between participants who completed the study and those who dropped out will be performed using the two-group *t* test for continuous variables and the Pearson chi-square test for categorical variables. Unadjusted within-group changes (from baseline to postintervention) will be assessed using the paired *t* test.

The primary method of analysis for physical activity, anthropometrics, and psychosocial measures, all of which will be measured at baseline, 6 months, 12 months, and 18 months (as illustrated in Table 1), will be mixed model repeated measures analyses. Other general linear mixed model techniques may also be used. These analyses will allow us to examine changes from baseline to follow-up (within-group changes) and differences between the study groups simultaneously, while also accounting for the group-by-time interaction as well as any covariates and interactions that are of scientific interest. An appropriate covariance matrix (eg, autoregressive or unstructured) will be selected based on the final data. The Tukey-Kramer multiple comparisons test will be used to determine specific pairwise differences for statistically significant main effects. Some of these models will include the stratification variable of county and confounding variables (as covariates) such as the baseline BMI category, age, gender, and education level. Some of the models that include physical activity as the dependent variable will be adjusted for wear time. Study variables that will be analyzed using these techniques include the change in minutes of moderate-to-vigorous physical activity. Pearson correlation analysis will be performed to assess the relationship between self-reported and measured physical activity.

Distributions of the aforementioned continuous variables will be examined for normality using box plots, normal probability plots, and the Kolmogorov-Smirnov test. Variables that are determined to deviate from a normal distribution will be log-transformed before statistical testing. Nonparametric tests (eg, the Wilcoxon rank-sum test and the Wilcoxon signed-rank test) may also be used to analyze nonnormally distributed data. All statistical tests will be two-sided. All analyses will be performed using SAS, and *P*<.05 will be deemed statistically significant.

Although a large amount of missing data is not expected for any of our study variables, a sensitivity analysis may be performed using alternative methods for handling missing data (such as multiple imputation) to assess the most appropriate approach based on the amount of missing data and effect sizes observed.

We will investigate potential mediators of the intervention effect (*social support*) using a multiple mediation approach in SPSS, in which all potential mediators are tested simultaneously, using a product of the coefficients method [52] with bootstrapped SEs (5000 samples with replacement). The interest is in estimating the path coefficients, effect sizes, and CIs rather than strict hypothesis testing.

Potential moderators and interactions will be assessed as follows. A variable will be considered a moderator if evidence exists of either qualitative or quantitative interaction with the intervention. We will use a similar analytic approach as in the primary aims; models will include the main effects of intervention (DIAL intervention vs control), the potential moderator (eg, education and neighborhood and environmental features), and the interaction between the two. Evidence of moderation exists if the coefficient of the interaction term is statistically different from zero.

Descriptive analyses of quantitative stakeholder acceptability survey items and content analyses of open-ended items from stakeholder acceptability surveys and focus group transcripts will be conducted to inform future efforts toward sustainability and large-scale dissemination in rural counties.

### Cost-effectiveness Analysis

If, as hypothesized, the DIAL intervention results in significantly greater increases in moderate-to-vigorous physical activity minutes, we will conduct a within-trial cost-effectiveness analysis [53-57] to determine if the DIAL intervention is cost-effective compared with no active intervention. Perspectives will be those of the health care sector, participants, and society. The time frame will be 6 months. We will estimate the DIAL implementation costs and participants’ medical and other costs that may be affected. Effectiveness will be measured by the change in moderate-to-vigorous physical activity minutes and QALY.

Implementation costs will include start-up and ongoing costs necessary to implement the DIAL intervention in other settings and will not include costs of intervention development and research activities (eg, consent process). Start-up costs will include time spent on training by trainers and intervention personnel, materials, space, and other supplies needed. To identify and value the DIAL intervention’s ongoing costs, we will develop process maps with intervention staff to identify all key processes (eg, supervision and orienting the participants to the IVR system, preparing, and IVR system tracking and maintenance) and the personnel involved in those processes, and develop a time tracking system to record the time spent in the identified processes. To reduce burden, this system will be used in random weeks by each intervention staff member. County coordinators will also complete time studies to estimate their time. Over the course of the study, we will select 1 week per month randomly for each intervention staff member. Data will then be annualized and combined with hourly wages and fringe benefits of the personnel to value annual personnel costs per activity. Costs of workbooks, handouts and other materials, phone and IVR system, office space, shipping, and others will be tracked and valued using project records or current market prices. Implementation cost data will be summed overall and by intervention-related categories, for example, IVR tracking and maintenance or feedback reporting. We will calculate the average DIAL intervention cost per participant and per minute moderate-to-vigorous physical activity increase.

The implementation costs of participants will include participation time costs, which will be captured with our surveys at baseline, 6 months, and 12 months. Survey questions will ask participants about the time spent reviewing intervention materials and calling into the IVR system and completing surveys and other intervention activities. These activities will also be tracked using the IVR system user data. Time costs will be valued using hourly wages and fringes based on average age and gender groups [58].

As improving physical activity has effects on well-being and potentially health care use, in the cost-effectiveness analysis, we will estimate medical costs for the DIAL intervention participants and control participants. At baseline, 6 months, and 12 months, we will use a health care utilization survey to capture information on physician and emergency room visits and hospitalizations [45]. To calculate medical costs, we will combine self-reported health care use and associated time and out-of-pocket costs and third-party payer unit costs. We will measure the cost of time spent exercising using the self-report 7-day PAR data and accelerometer data. All time costs will be valued using hourly wages and fringes based on average age and gender groups [58].

Medical and other costs will be added to the implementation costs. Incremental cost-effectiveness ratios (ICERs) will be calculated as the average net cost per minute of moderate-to-vigorous physical activity. In a previous study, physical activity interventions had ICERs of US $0.05-US $0.15 per moderate-to-vigorous physical activity minute [59,60]. We will also calculate the ICERs per QALY gained. QALYs will be calculated over a 6-month follow-up period using utility weights derived from the EuroQol-5D [46]. To determine if the DIAL intervention is cost-effective compared with no active intervention, ICERs will be compared with the commonly used threshold of US $50,000-US $100,000 per QALY [61].

To examine uncertainty, we will sample the replacement costs and outcomes from the two trial arms and calculate the mean costs and outcomes for each bootstrap sample, repeating the procedure 1000 times. Differences in costs and outcomes between the two groups from each sample will be plotted in a cost-effectiveness plane [61,62]. ICERs will be obtained for each sample, and confidence limits around the ICER will be obtained by taking the values at the 5th and 95th percentile of the distribution. Analyses will be repeated to examine the uncertainty around data inputs, such as hourly wages or medical care costs. In addition, we will construct an acceptability curve by considering the proportion of bootstrap replications for which the ICER falls below the possible thresholds of cost per QALY [63].

## Results

Start-up activities (staff hiring and training, ordering supplies, and equipment) have been completed. Although the suspension of nonessential research activities at UAB in March 2020 due to COVID-19 delayed the start of the clinical trial, this time was used to refine intervention and assessment protocols to address new health and safety concerns and further beta test and improve the automated telephone counseling system. Participant recruitment and data collection began in September 2020 and is ongoing. In Dallas County, 43 participants completed baseline assessments at the local Young Men’s Christian Association in September 2020, and all participants have been randomized to the study arm. In addition, all 43 Dallas County participants have completed their 3-month surveys. In Sumter County, 42 participants completed baseline assessments at a local community center in November 2020. Moreover, 51 participants completed baseline assessments in Greene County at a local high school in early February 2021. Baseline assessments for the next county (Marengo) are projected to begin mid-March 2021, followed by 3-month surveys in Sumter County, and 6-month assessments in Dallas County.

## Discussion

### Principal Findings

This study will test the efficacy of the DIAL intervention versus a wait-list control condition for increasing physical activity in the Deep South region of the United States, an area with high rates of physical inactivity and related cancer disparities. To our knowledge, this is the first study to implement an IVR system–supported physical activity intervention for underserved (minority, rural) populations. Past IVR-based physical activity interventions were conducted in largely White and well-educated [6] populations. Some studies relied on unidirectional IVR system calls (ie, system-initiated) [7,8], whereas others involved bidirectional IVR system calls [6,9] (system and participant initiated), as in this study. Promising findings, for example, increases in physical activity [6,8], increases in muscle strength, improvements in balance [7], and decreases in one-mile walk time [9], bode well for the success of the current efforts. Past physical activity–related IVR system–based studies [6-9] were grounded in evidence-based behavioral science theory (ie, SCT and/or Transtheoretical Model) and were found to be effective for increasing physical activity. Some past studies involved a short intervention duration (ie, 12 weeks) [8,9]. Studies conducted over longer periods (ie, 12 months) found relative maintenance of physical activity over the course of the intervention [6,7]. This study will extend this line of research to an at-risk sample of rural, mostly minority adults in the Deep South and extend the follow-up to 18 months.

As for limitations, at 12 months, we will not be able to compare the DIAL intervention arm to a true control arm. As previously mentioned, a 12-month wait-list was proposed but stakeholders felt that the 12-month waiting period was too long and could consequently result in a lack of interest in the study, reduced engagement, and/or drop out among wait-list control participants. Finally, we had to reduce our survey battery and used short-form versions in response to concerns regarding participant burden. Despite the established validity of these measures, using the short versions could result in potentially less validation through assessments of these psychosocial constructs.

The strengths of the ongoing study include the randomized controlled design, hybrid (ie, efficacy combined with implementation outcomes), multilevel intervention, evidence-based theoretical framework, collection of built environment data, and assessment of barriers to and facilitators of future broader dissemination and implementation of the DIAL program with rural county coordinators and UAB O’Neal CCC COE. Another key strength is the use of high-reach, low-cost, technology-supported strategies for addressing rural health disparities. Tracking intervention costs will allow us to comment further on the cost-effectiveness of this approach. We will assess physical activity objectively using accelerometers as opposed to relying solely on subjective, self-reported data, unlike previous studies [6,8,9]. Other areas in which this work moves the field forward include (1) assessing long-term outcomes (12-18 months) and (2) determining the effectiveness and convenience of scheduled calls made by the out-DIAL program (as opposed to solely participant-initiated calls).

### Conclusions and Clinical Implications

The findings from this study will help establish the efficacy of IVR system–supported physical activity promotion strategies for underserved rural regions. Moreover, these findings have implications for health care providers and public health practice for physical activity promotion when barriers such as distance, transportation, and lack of staff hinder face-to-face visits and interaction. Future directions include (1) working with stakeholders to address identified barriers to implementation and sustainability in rural counties, (2) pursuing further large-scale dissemination of the DIAL intervention, and (3) addressing built environment concerns in the rural communities through policy advocation and implementation, based on RALA findings.

## Appendix

Multimedia Appendix 1 Peer review by the Center for Scientific Review Special Emphasis Panel.

## Abbreviations

DIAL: Deep South Interactive Voice Response System–Supported Active Lifestyle
ICER: incremental cost-effectiveness ratio
IRB: Institutional Review Board
IVR: interactive voice response
PAR: physical activity recall
QALY: quality-adjusted life year
RALA: Rural Active Living Assessment
SCT: Social Cognitive Theory
UAB O’Neal CCC COE: University of Alabama at Birmingham O’Neal Comprehensive Cancer Center Office of Community Outreach and Engagement
