# Supplementary material for: Design and Rationale for the Deep South Interactive Voice Response System–Supported Active Lifestyle Study: Protocol for a Randomized Controlled Trial
Source: JMIR Res Protoc. 2021 May 25;10(5):e29245. doi: 10.2196/29245 (PMC8188314; doi:10.2196/29245)
Supplement: Multimedia Appendix 1 [file resprot_v10i5e29245_app1.docx]

**PROGRAM CONTACT:**

**Frank Perna (240) 276-6782**

[**pernafm@mail.nih.gov**](mailto:pernafm@mail.nih.gov)

**SUMMARY STATEMENT**

**( Privileged Communication )**

***Release Date:* 03/18/2019**

***Revised Date:***

**Principal Investigator PEKMEZI, DOROTHY W**

***Application Number:* 1 R01 CA233550-01A1**

**Applicant Organization: UNIVERSITY OF ALABAMA AT BIRMINGHAM**

***Review Group:* ZRG1 RPHB-W (55)**

**Center for Scientific Review Special Emphasis Panel**

**PAR Panel: Developing and Testing Interventions for Health-Enhancing Physical Activity**

***Meeting Date:* 03/04/2019 *RFA/PA:* PAR18-324**

***Council:* MAY 2019 *PCC:* H6HP**

***Requested Start:* 07/01/2019**

***Dual IC(s):* MD**

***Project Title:* Testing scalable, IVR-supported cancer prevention interventions in the rural Alabama Black Belt**

***SRG Action:* Impact Score:18 Percentile:2 #**

***Next Steps:* Visit https://grants.nih.gov/grants/next_steps.htm**

**Human Subjects: 30-Human subjects involved - Certified, no SRG concerns Animal Subjects: 10-No live vertebrate animals involved for competing appl.**

**Gender: Minority: Children:**

**1A-Both genders, scientifically acceptable**

**1A-Minorities and non-minorities, scientifically acceptable 3A-No children included, scientifically acceptable**

| **Project**  **Year** | **Direct Costs**  **Requested** | **Estimated**  **Total Cost** |
| --- | --- | --- |
| **1** | **499,936** | **729,679** |
| **2** | **499,950** | **729,700** |
| **3** | **499,950** | **729,700** |
| **4** | **499,950** | **729,700** |
| **5** | **499,950** | **729,700** |

**TOTAL**

**2,499,736**

**3,648,479**

**NEW INVESTIGATOR**

# 1R01CA233550-01A1 Pekmezi, Dorothy NEW INVESTIGATOR

**RESUME AND SUMMARY OF DISCUSSION:** This application proposes a fully powered randomized controlled trial using an Interactive Voice Response (IVR) system-supported telehealth intervention to promote physical activity for cancer risk reduction in the Deep South. The project is strongly grounded in theory and positive preliminary studies that support rigor and potential success. The population is hard to reach, but the technology-driven strategies would help extend the reach of ongoing community health worker efforts into rural Black Belt communities. The effective and excellent intervention could have a significant impact. The strong investigative team is led by an excellent New Investigator. The multi-level intervention design is strengthened in this resubmission to include personal, interpersonal and community levels and the addition of an expert in environmental factors. The resubmission also addressed the concern about the association between physical activity and cancer risk. The panel was very enthusiastic about the much-improved application, raising only minor concerns during discussion. It was noted that the logistics and scalability of the community component appeared weaker than the personal and interpersonal components, and there was some lack of detail on the cost-effective analysis and IVR dose/intensity. Overall, this well-designed physical activity intervention could contribute significantly to addressing health problems in an underserved and high risk population.

**DESCRIPTION (provided by applicant):** Regular physical activity (PA) is linked to lower risk for several cancers (breast, colon, endometrial). Yet, most Americans are inactive, particularly in the Deep South. Cancer incidence/mortality is also generally higher in this region, with underserved (rural, African American) populations reporting even less PA and disproportionate cancer burden. Factors related to culture, distance from PA facilities, income, literacy, and Internet connectivity in the Deep South may limit access to PA information/resources and contribute to existing cancer disparities.

Telephone-supported interventions have shown success in increasing PA, do not require clinic visits, literacy, or costly technology, and thus may represent a promising strategy for promoting PA for cancer risk reduction in the Deep South, especially when automated with Interactive Voice Response (IVR) systems for improved cost- effectiveness and reach. Thus, we adapted an existing IVR system, used in past HIV studies, for PA promotion and cancer prevention in the Deep South through extensive literature review and formative research [11 focus groups on PA intervention needs/preferences with Deep South Network For Cancer Control community health advisors (CHAs) and community members]. A pilot trial of the resulting Deep south IVR-supported Active Lifestyle (DIAL) intervention! (R03CA177538) found high retention and participant satisfaction at 12 weeks. Moreover, DIAL produced larger increases in MVPA (42.5 more min/week) from baseline to 12 weeks than a waitlist control, along with significantly greater improvements in PA self-regulation and social support. Results have informed intervention enhancements in preparation for scale up and dissemination in rural counties (using IVR-initiated calls and wristbands to facilitate adherence; further targeting unchanged Social Cognitive Theory constructs; incorporating more interpersonal, community/organizational and policy level strategies for increased encouragement and accountability and to assess and address built environment). The current study will involve a fully powered randomized controlled trial of the refined, multi-level 12-month DIAL intervention with a waitlist control condition (N=240 rural, mostly African American adults). Recruitment will be led by Deep South Network for Cancer Control CHAs in Black Belt counties (involved in project since initial formative research). Primary aims include examining arm differences in changes in moderate intensity or greater aerobic physical activity from baseline to 6 and 12 months. Exploratory aims include examining intervention effects on physical performance and psychosocial variables; changes in physical activity from 12- 18 months; intervention costs; potential mediators (social support from family, friends, CHAs) and moderators of treatment efficacy (neighborhood/ environmental features); and potential barriers/facilitators to widespread implementation of DIAL intervention in rural Black belt counties by Deep South Network for Cancer Control.

**PUBLIC HEALTH RELEVANCE**: This proposal tests a multi-level, interactive voice response system- supported physical activity intervention in populations at high risk for sedentary behavior (rural, mostly minority residents in the Deep South). This line of research will help extend the reach and sustainability of ongoing community health worker efforts by the Deep South Network for Cancer Control in rural Black Belt communities and thereby address related cancer disparities.

# CRITIQUE 1

Significance: 1

Investigator(s): 1

Innovation: 4

Approach: 1

Environment: 1

**Overall Impact:** This is a revised application of a highly significant and potentially highly impactful R01 application for a well-powered randomized trial to test the efficacy of an automated phone-based system for increasing physical activity in the deep south where inactivity and cancer prevalence is high. Telephone-supported interventions have shown success in increasing PA low cost and may be a promising strategy for promoting PA in the Deep South. This application proposes an Interactive Voice Response (IVR) system-supported telehealth intervention to promote physical activity for cancer risk reduction in the Deep South. This team has adapted an existing IVR system for PA promotion in the Deep South [Deep south IVR-supported Active Lifestyle - DIAL] and found high retention, participant satisfaction, increase in MVPA over 12 weeks, and improvements in PA self-regulation and social support. Results have informed intervention enhancements in preparation for scale up and dissemination in rural counties (targeting Social Cognitive Theory constructs; incorporating more interpersonal, community/organizational and policy level strategies for increased encouragement and accountability and to assess and address built environment). The current study will involve a fully powered randomized controlled trial of the refined, multi-level 12 month DIAL intervention with a waitlist control condition (N=240 rural, mostly African American adults). Recruitment will be led by Deep South Network for Cancer Control CHAs in Black Belt counties. Primary aims include examining arm differences in changes in moderate intensity or greater aerobic physical activity from baseline to 6 and 12 months. Exploratory aims include examining intervention effects on physical performance and psychosocial variables; changes in physical activity from 1218 months; intervention costs; potential mediators (social support from family, friends, CHAs) and moderators of treatment efficacy (neighborhood/ environmental features); and potential barriers/facilitators to widespread implementation of DIAL intervention in rural Black belt counties by Deep South Network for Cancer Control.

# Significance:

**Strengths**

- - Physical inactivity and cancer are both major public health problems that are particularly alarming in the Deep South.
  - The proposed intervention is innovative and has potential for effectiveness and reach throughout remote communities.
  - Deep South communities tend to be rich in minorities and underserved, remote populations.
  - Rural areas are hard to reach; thus a mobile phone-based system could have high impact on breaking down barriers.

# Weaknesses

- - None noted.

# Investigator(s):

**Strengths**

- - Research team has a solid history of collaboration and studies that support this application. Investigators also have the relevant expertise in physical activity research (Pekmezi, Demark- Wahnefried); technology-supported, individually-tailored lifestyle interventions (Pekmezi, Demark-Wahnefried); IVR system management and programming (Thirumalai), minority/rural recruitment and retention (Hardy; Pekmezi); statistics (Oster); and cost analyses (Pisu) to carry out the proposed research plan.
  - Since the initial submission, an expert in environmental determinants of obesity in the Deep South (Dr. Baskin) and Rural Active Living Assessment tools (RALA) has been added. This will allow the investigators to describe/analyze environmental/ neighborhood data by county, which may serve as potential moderators of intervention efficacy.

# Weaknesses

- - None noted.

# Innovation:

**Strengths**

- - Adapted IVR tool from HIV research for use in physical activity promotion.

# Weaknesses

- - Not entirely new or high risk. Technology and methods already developed.

# Approach:

**Strengths**

- - Large, randomized rigorous design, single blind.
  - Objectively measured activity.
  - Delayed intervention wait-list control for replication of intervention effects.
  - Well connected to community for dissemination and implementation through Deep South Network for Cancer Control county coordinators and community health workers in recruitment, intervention, and assessment to enhance community buy-in and sustainability and discern challenges/ facilitators to future large-scale implementation of such technology-supported interventions in rural counties.
  - Strong preliminary data and track record for recruitment and retention in these communities.
  - Assessments and workflow highly organized and efficient. Intervention strongly grounded in theory and best practice for physical activity promotion.
  - Comprehensive well-designed data analysis plan.
  - Robust cost-effectiveness analyses are planned.
  - Potential limitations and pitfalls and future directions are anticipated.
  - A concern from the initial submission of this proposal was that it lacked a multi-level intervention. The investigators have now addressed this major concern. The intervention will address individual, interpersonal, community, organizational, and policy levels of the socio- ecological model.
  - The prior review of the first submission also raised concern for the relevance to cancer prevention. The revised proposal now adequately addressed this concern in the Significance section of the proposal.

# Weaknesses

- - None noted.

# Environment:

**Strengths**

- - Very strong academic working, collaborative environment with robust community connections and networks.

# Weaknesses

- - - None noted.

# Study Timeline:

**Strengths**

- - - Realistic with built in flexibility over 5 years.

# Weaknesses

- - - None noted.

# Protections for Human Subjects:

Acceptable Risks and/or Adequate Protections

Data and Safety Monitoring Plan (Applicable for Clinical Trials Only):

- - - Acceptable and Appropriate

# Vertebrate Animals:

- - - Not Applicable (No Vertebrate Animals)

# CRITIQUE 2

Significance: 2

Investigator(s): 1

Innovation: 3

Approach: 2

Environment: 1

**Overall Impact:** This is an outstanding proposal that responded to earlier critiques to strengthen the investigative team and approach. The focus is on a high-need region and population (rural, primarily African American adults) that experiences disparities in cancer outcomes and cancer preventing physical activity. The investigative team is led by a new investigator with a strong track record of intervention with the population and includes a balance of earlier and later career investigators—each with a specific, necessary, and well-defined role. The approach includes a practical IVR intervention that is combined with community-based strategies and supports. Cost considerations are a strength of this proposal as is the community-engaged research approach. A robust design with validated assessments, including objectively assessed physical activity, health care utilization, and quality of life

will allow for a number of hypotheses to be tested including and expanding beyond the primary aim. The innovative use of high frequency IVR and incentivizing initial call completion are innovative components of the study. A few minor weaknesses are noted in the areas of assessing maintenance of behavior change without a comparison group that has not received intervention (though this is balanced by allowing waitlist control participants to engage in the intervention after 12 months rather than waiting 18 months), cost analysis not clearly articulating community components of the intervention, and limited focus on activities to re-engage participants with the IVR system.

# Significance:

**Strengths**

- - Rural African-Americans participate in lower levels of physical activity wait compared to other racial groups and experience a disproportionate incidence of cancer.
  - In addition to challenges experienced by other rural populations relative to geographic factors cultural factors need to be addressed when promoting physical activity.
  - Clear link is described between physical activity and cancer risk and the disparities for African- Americans in the deep south related to physical activity, cancer incidence, and cancer mortality.
  - Telephone-based strategies have the potential for broader reach in this area and automated telephone counseling maybe a promising approach for this audience.
  - Strong preliminary work suggests that the IVR application for an intervention with this population shows promise.

# Weaknesses

- - There have been IVR studies conducted for PA promotion in older adults and in primarily rural areas in Appalachia that would provide some additional context—especially related to the high versus lower frequency of calls. These could provide additional support for the need to test higher intensity versions of this type of intervention. (minor weakness)

# Investigator(s):

**Strengths**

- - Principal investigator is a new investigator who has completed a number of physical activity trials for underserved populations and has pilot tested the proposed intervention. She also has strong expertise in theory-based physical activity interventions and expertise in mixed methods and qualitative research to refine and improve intervention content and design.
  - Investigative team is a nice balance of earlier and later career investigators and though it includes a large number of investigators, each has a specific role defined for the project.

# Weaknesses

- - None noted

# Innovation:

**Strengths**

- - IVR is moderately innovative.
  - The combination of objectively assessed physical activity through wristbands as a component of the IVR feedback system is innovative.
  - The use of modest financial incentives for call completion is an innovative approach to address retention in the high volume of calls. The modest nature of these incentives reduces the likelihood of participants completing intervention sessions and physical activity solely to receive

approximately $15 per month (the amount possible with complete compliance)—though it should provide a nudge towards completion.

# Weaknesses

- - Many of the areas described in the innovation section are restatement of either the significance of the project (e.g., theory-based approach) or approach features (e.g., cost analyses; minor weakness).

# Approach:

**Strengths**

- - Randomized controlled trial with shorter term effectiveness and maintenance assessments.
  - Theory-based intervention development combined with community and participant feedback.
  - Assessment of mediators and moderators in examining relationships between the intervention and primary outcome.
  - The assessment of costs and cost-effectiveness evaluation.
  - Strong partnerships with the Deep South Network for cancer control provides some basis for the likelihood of future dissemination and implementation.
  - The partnership with the network provides a basis to expect effective recruitment and retention within the trial.
  - Addressing issues that are common among lower income and rural population to improve accessibility of study participation such as providing transportation and childcare expenses for assessments is a real strength.
  - Taking assessments into the community for ease of completion for participants into reduce burden is also strength.
  - Combining seven-day PA recalls and accelerometer data call provide opportunities to understand type, time, intensity, and duration of physical activity.
  - Waitlist control participants will receive intervention following the 12-month assessment. This is a strength in that it provides an opportunity for all study participants to benefit within a year of initiating participation (however, it also reduces the ability to have a comparison condition for the 18 month follow up; minor weakness).
  - Tailoring based on progress using a more objective assessment of physical activity.
  - Counseling messages in the intervention will be based on social cognitive theory.
  - Connecting participants to community and organizational level resources through the community health advisers is an excellent improvement on this trial.
  - Focus on policy level approaches later in the trial to address community issues that reduce the likelihood of physical activity.

# Weaknesses

- - The use of IVR is it in much higher frequency compared to other physical activity interventions that have used IVR. However, this intensity of calls appears to have been tested in the preliminary work and was acceptable to the participants and scaling back of the number of calls per week after the first three months seems appropriate and makes this, overall, a minor weakness.
  - There’s no description of what will be done to support participants who disengage from the IVR system. Will there be possibility to receive a scaled-back frequency of calls that focuses primarily on counseling components?
  - Cost assessment does not seem to include all levels of intervention, in particular the community health adviser engagement and policy and advocacy efforts do not seem to be included.

# Environment:

**Strengths**

- - The environment is well suited to support this research.

# Weaknesses

- - None noted.

# Study Timeline:

**Strengths**

- - The timeline is well thought out and is realistic in the time needed to complete the recruitment (30 months) with the possibility of completing enrollment early.

# Weaknesses

- - None noted.

# Protections for Human Subjects:

Acceptable Risks and/or Adequate Protections

Data and Safety Monitoring Plan (Applicable for Clinical Trials Only): Acceptable

# Inclusion of Women, Minorities and Children:

- Sex/Gender: Distribution justified scientifically
- Race/Ethnicity: Distribution justified scientifically
- For NIH-Defined Phase III trials, Plans for valid design and analysis: Not applicable
- Inclusion/Exclusion of Children under 18: Excluding ages <18; justified scientifically

# Vertebrate Animals:

Not Applicable (No Vertebrate Animals)

# Biohazards:

Not Applicable (No Biohazards)

# Resubmission:

- Investigators did a nice job highlighting the multiple levels of the proposed intervention including efforts to examine potential policy changes to take the intervention to scale.
- Investigators also included significant detail on the relationship between moderate and vigorous physical activity with cancer risk.
- Investigative team was strengthened to include consideration of environmental determinants.

# Budget and Period of Support:

Recommend as Requested

# CRITIQUE 3

Significance: 1

Investigator(s): 1

Innovation: 2

Approach: 2

Environment: 1

**Overall Impact:** The investigators propose an IVR-based intervention to promote PA among rural, primarily African American communities in the Deep South. The project also has a cancer prevention focus, since physical inactivity is associated with increased cancer risk. A critique of the initial submission was related to lack of a strong tie between the intervention and CA risk. The authors have addressed this critique by adding more information about the association between PA and cancer risk. I think there is still a little bit of a disconnect between the specific target of CA risk and the general intervention of PA. However, given that the focus of this funding mechanism is not CA-specific, I think this is acceptable. PA has benefits for many other health conditions that have a higher prevalence and severity in the communities on which the investigators will focus. Therefore, I would encourage the investigators to collect robust data on outcomes of relevance to other chronic health conditions as well. Another critique was related to the primarily individual level within the socioecological model. This has also been addressed in the revised submission to incorporate interpersonal components and some environmental / community aspects. The latter are still not an emphasis of the intervention, but I think there is adequate attention given to multiple intervention levels, and this could be built upon in future interventions. Overall the proposal addresses a significant health problem and an underserved / high risk population. The proposed intervention is scalable and there are novel aspects to it. The methods of the study are rigorous, and there is a very strong investigative team. I think this is a very strong proposal.

# Significance:

**Strengths**

- - The proposed study addresses an underserved community with higher risk for cancer and other health conditions.
  - There is a lack of scalable interventions for this patient group.

# Weaknesses

- - With respect to CA control as a main focus of this type of intervention, although there are clear data showing a link between PA and CA, if the primary objective is to CA control, I think other approaches are likely to be more potent (e.g., enhanced screening, more comprehensive behavioral strategies including weight loss).

# Investigator(s):

**Strengths**

- - Dr. Pekmezi has ample relevant experience in PA interventions for underserved interventions, had led the pilot work leading to this proposal, and has been collaborating with the Deep South Network for Cancer Control.
  - Co-investigators are also very experienced and have complementary expertise in trials of PA interventions for CA survivors and other patient groups (Demark-Wahnefried), biostatistics

(Oster), economics (Pisu), health informatics (Thirumalai), and minority health behaviors and disparities (Baskin).

# Weaknesses

- - None noted.

# Innovation:

**Strengths**

- - Use of IVR for intervention delivery, particularly in this population.
  - Combining PA monitoring (wristbands) with the IVR intervention.
  - Use of small monetary incentive to promote PA reporting.

# Weaknesses

- - None noted.

# Approach:

**Strengths**

- - Pilot data support the intervention approach.
  - Overall rigorous and well described methods.
  - Multi-faceted recruitment approach.
  - Attention to potential participant barriers such as payment for transportation & child care.

# Weaknesses

- - The newly added intervention components in places feel like “add-ons” that are not entirely tied into the individual level intervention, and they are not as well developed.
  - It is not clear how & when the newly added CHA activities (e.g. guidance in problem solving) will happen.

# Environment:

**Strengths**

- - Environment has the needed resources to support a project of this type.
  - Established collaboration with the Deep South Network for Cancer Control.

# Weaknesses

**Study Timeline:**

**Strengths**

- - Overall reasonable timeline.

# Weaknesses

- - No significant concerns.

# Protections for Human Subjects:

Acceptable Risks and/or Adequate Protections

Data and Safety Monitoring Plan (Applicable for Clinical Trials Only):

Acceptable

# Inclusion of Women, Minorities and Children:

- - Sex/Gender: Distribution justified scientifically
  - Race/Ethnicity: Distribution justified scientifically
  - For NIH-Defined Phase III trials, Plans for valid design and analysis:
  - Inclusion/Exclusion of Children under 18: Excluding ages <18; justified scientifically

# Vertebrate Animals:

Not Applicable (No Vertebrate Animals)

# Biohazards:

Not Applicable (No Biohazards)

# Resubmission:

- - Overall, I believe the investigators have responded adequately to the initial reviews. One critique of the initial submission was related to lack of a strong tie between the intervention and CA risk. The authors have addressed this critique by adding more information about the association between PA and cancer risk. I think there is still a little bit of a disconnect between the specific target of CA risk and the general intervention of PA. However, given that the focus of this funding mechanism is not CA-specific, I think this is acceptable. PA has benefits for many other health conditions that have a higher prevalence and severity in the communities on which the investigators will focus. Therefore, I would encourage the investigators to collect robust data on outcomes of relevance to other chronic health conditions as well. Another critique was related to the primarily individual level within the socioecological model. This has also been addressed in the revised submission to incorporate interpersonal components and some environmental / community aspects. The latter are still not an emphasis of the intervention, but I think there is adequate attention given to multiple intervention levels, and this could be built upon in future interventions.

# Resource Sharing Plans:

Acceptable

# Budget and Period of Support:

Recommend as Requested

# THE FOLLOWING SECTIONS WERE PREPARED BY THE SCIENTIFIC REVIEW OFFICER TO SUMMARIZE THE OUTCOME OF DISCUSSIONS OF THE REVIEW COMMITTEE, OR REVIEWERS' WRITTEN CRITIQUES, ON THE FOLLOWING ISSUES:

**PROTECTION OF HUMAN SUBJECTS: ACCEPTABLE INCLUSION OF WOMEN PLAN: ACCEPTABLE INCLUSION OF MINORITIES PLAN: ACCEPTABLE**

**INCLUSION OF CHILDREN PLAN: ACCEPTABLE**

**COMMITTEE BUDGET RECOMMENDATIONS: The budget was recommended as requested.**

Footnotes for 1 R01 CA233550-01A1; PI Name: Pekmezi, Dorothy W

# Ad hoc or special section application percentiled against "Total CSR" base.

NIH has modified its policy regarding the receipt of resubmissions (amended applications). See Guide Notice NOT-OD-14-074 at <http://grants.nih.gov/grants/guide/notice-files/NOT-OD-> 14-074.html. The impact/priority score is calculated after discussion of an application by averaging the overall scores (1-9) given by all voting reviewers on the committee and multiplying by 10. The criterion scores are submitted prior to the meeting by the individual reviewers assigned to an application, and are not discussed specifically at the review meeting or calculated into the overall impact score. Some applications also receive a percentile ranking. For details on the review process, see [http://grants.nih.gov/grants/peer_review_process.htm#scoring.](http://grants.nih.gov/grants/peer_review_process.htm#scoring)

**MEETING ROSTER**

**Center for Scientific Review Special Emphasis Panel CENTER FOR SCIENTIFIC REVIEW**

**PAR Panel: Developing and Testing Interventions for Health-Enhancing Physical Activity**

**ZRG1 RPHB-W (55) 03/04/2019**

**Notice of NIH Policy to All Applicants:** Meeting rosters are provided for information purposes only. Applicant investigators and institutional officials must not communicate directly with study section members about an application before or after the review. Failure to observe this policy will create a serious breach of integrity

in the peer review process, and may lead to actions outlined in NOT-OD-14-073 at https://grants.nih.gov/grants/guide/notice-files/NOT-OD-14-073.html and NOT-OD-15-106 at https://grants.nih.gov/grants/guide/notice-files/NOT-OD-15-106.html, including removal of the application from immediate review.

**CHAIRPERSON(S)** SAELENS, BRIAN E, PHD PROFESSOR

CENTER FOR CHILD HEALTH, BEHAVIOR AND DEVELOPMENT

SEATTLE CHILDREN'S HOSPITAL RESEARCH INSTITUTE UNIVERSITY OF WASHINGTON

SEATTLE, WA 98101

**MEMBERS**

ALLEN, KELLI D., PHD PROFESSOR

DIVISION OF RHEUMATOLOGY, ALLERGY, AND IMMUNOLOGY

SCHOOL OF MEDICINE

UNIVERSITY OF NORTH CAROLINA AT CHAPEL HILL CHAPEL HILL, NC 27599

BEETS, MICHAEL W, PHD

ESTABROOKS, PAUL, PHD PROFESSOR

DEPARTMENT OF HEALTH PROMOTIONS, SOCIAL & BEHAVIORAL HEALTH COLLEGE OF PUBLIC HEALTH

UNIVERSITY OF NEBRASKA MEDICAL CENTER OMAHA, NE 68198

JACKSON, ELIZABETH A., MD PROFESSOR

DIVISION OF CARDIOVASCULAR DISEASE UNIVERSITY OF ALABAMA BIRMINGHAM BIRMINGHAM, AL 35294

LI, FUZHONG, PHD

SENIOR RESEARCH SCIENTIST OREGON RESEARCH INSTITUTE EUGENE, OR 97403

PROFESSOR PEREIRA, MARK A, PHD

DEPARTMENT OF EXERCISE SCIENCE ARNOLD SCHOOL OF PUBLIC HEALTH UNIVERSITY OF SOUTH CAROLINA COLUMBIA, SC 29208

CADMUSBERTRAM, LISA ANNE, PHD

PROFESSOR

DIVISION OF EPIDEMIOLOGY AND COMMUNITY HEALTH UNIVERSITY OF MINNESOTA MINNEAPOLIS, MN 55454

ASSISTANT PROFESSOR PRELIP, MICHAEL LEE, DPA

DEPARTMENT OF KINESIOLOGY UNIVERSITY OF WISCONSIN-MADISON MADISON, WI 53706

CONROY, DAVID E., PHD PROFESSOR

DEPARTMENT OF KINESIOLOGY

COLLEGE OF HEALTH AND HUMAN DEVELOPMENT PENNSYLVANIA STATE UNIVERSITY

UNIVERSITY PARK, PA 16802

DIAZ, KEITH M, PHD ASSISTANT PROFESSOR DEPARTMENT OF MEDICINE DIVISION OF CARDIOLOGY COLUMBIA UNIVERSITY NEW YORK, NY 10032

PROFESSOR

DEPARTMENT OF COMMUNITY HEALTH SCIENCES SCHOOL OF PUBLIC HEALTH

UNIVERSITY OF CALIFORNIA LOS ANGELES LOS ANGELES, CA 90095

ROSENKRANZ, RICHARD R., PHD ASSOCIATE PROFESSOR

DEPARTMENT OF FOOD, NUTRITION, DIETETICS AND HEALTH

KANSAS STATE UNIVERSITY MANHATTAN, KS 66506

SACHECK, JENNIFER, PHD

SENIOR PROFESSOR OF PREVENTION AND WELLNESS DEPARTMENT OF EXERCISE AND NUTRITION SCIENCES MILKEN INSTITUTE SCHOOL OF PUBLIC HEALTH

THE GEORGE WASHINGTON UNIVERSITY WASHINGTON, DC 20052

SALVATORE, ALICIA LINK, PHD ASSISTANT PROFESSOR

HUDSON COLLEGE OF PUBLIC HEALTH THE UNIVERSITY OF OKLAHOMA HEALTH SCIENCES CENTER OKLAHOMA CITY, OK 73126

STEWART, KERRY J., EDD PROFESSOR OF MEDICINE

CLINICAL/RESEARCH EXERCISE PHYSIOLOGY JOHNS HOPKINS UNIVERSITY

BALTIMORE, MD 21224

TUDOR-LOCKE, CATRINE E., PHD PROFESSOR

DEPARTMENT OF KINESIOLOGY UNIVERSITY OF MASSACHUSETTS AMHERST, MA 01000

WOLIN, KATHLEEN Y, SCD PRINCIPAL

COEUS HEALTH, LLC CHICAGO, IL 60654

**SCIENTIFIC REVIEW OFFICER**

NI, WEIJIA, PHD

CHIEF/SCIENTIFIC REVIEW OFFICER

RISK, PREVENTION AND HEALTH BEHAVIOR INTERGRATED REVIEW GROUP

CENTER FOR SCIENTIFIC REVIEW NATIONAL INSTITUTES OF HEALTH BETHESDA, MD 20892

**EXTRAMURAL SUPPORT ASSISTANT**

FAYEMIWO, TOLU

EXTRAMURAL SUPPORT ASSISTANT CENTER FOR SCIENTIFIC REVIIEW NATIONAL INSTITUTES OF HEALTH BETHESDA, MD 20892

Consultants are required to absent themselves from the room during the review of any application if their presence would constitute or appear to constitute a conflict of interest.
